# Supplementary material for: Screening of aspiration pneumonia using the modified Mallampati classification tool in older adults
Source: PLoS One. 2024 May 10;19(5):e0302384. doi: 10.1371/journal.pone.0302384 (PMC11086910; doi:10.1371/journal.pone.0302384)
Supplement: Attachment — Submitted filename: plos review.docx [file pone.0302384.s001.docx]

Date: 12/24/2023

PLOS ONE

Review report

Title: Oropharyngeal morphology (modified Mallampati classification) could be a useful screening tool for the risk of pneumonia in the older adults

I. General comments

I would like to express my sincere appreciation for the study you have conducted. The work you have done is very substantial for evidence-based public health intervention, particularly in the context of the aged population. However, I would like to suggest that some revisions are necessary to address editorial and grammatical errors, as well as specific comments in each section.

II. Specific comments

1. Title

The title should be like “Screening of Aspiration Pneumonia using the Modified Mallampati Classification Tool in Older Adults”.

2. Abstract

The introduction part of the abstract should be rewrited. It is so long. Make it short and concise. From lines 19 to 21 is not clear: “We hypothesized that changes in the oropharyngeal morphology and hyoid bone position may lead to the evaluation of swallowing function, which in turn determines the risk of aspiration pneumonia”. Please rewrite it as ‘Changes in the oropharyngeal morphology and hyoid bone position may lead to the risk of aspiration pneumonia’. Lines 30-32: “The lower the classification (small size of the base of the tongue relative to the oropharyngeal cavity), the higher was the incidence of aspiration pneumonia.” This statement is not clear. Please rephrase or delete it.

3. Introduction

Dear authors, in the introduction section, lines 38 to 43 have no references. This is not acceptable in a scientific work. Please add citations in each statement unless interlinked. Similarly lines 47 to 63 have no references. Dear authors, you only cite 2 references in the whole section of the introduction. Please take more time, you have to cite every piece of evidence that you wrote. Please rephrase each paragraph. It needs English language revision. For instance, lines 45 to 46: “Thus, there is an urgent need to manage aspiration pneumonia in an aging society.” Should be moved to the last paragraph of the ‘introduction’ section.

4. Methods

Dear authors, please add study area/setting, study period and sample size calculation sub-headings. Please move ethical declaration statements with sub heading “ethical approval” (Lines 71 to 76) to the end of ‘methods’ section or at the end of the manuscript before the ‘references’ section. Please separately state your inclusion and exclusion criteria. I think you need to add “operational definition” subheading. For example: menton, laryngeal prominence and validity and reliability of your measurements. Please consult native speaker of English language for grammar and punctuation errors.

5. Results

Dear authors no need of this statement “The characteristics of the participants are presented in Table 1.” Instead please cite table 1 in parenthesis at the end of this paragraph. Lines 140 to 141 should be rewrite as ‘The median age of participants (men: 92, women: 99) was 79.3 years with a range of 65 to 98 years’. Dear authors, please rewrite lines 142 to 144 as ‘About 152 (79.6%) participants had been evaluated for saliva swallowing tests more two times.’ Please continue like this …..No need of writing lower frequencies or percentages in the table. Focus on pertinent findings.

Dear authors please delete lines 247 to 248 and cite table 5 in parentheses at the end of this paragraph.

6. Discussions

Dear authors, please delete “We believe that this is the first study to examine the relationship between the Mallampati classification and the risk of aspiration pneumonia”. No need of this statement rather if possible it is better to add a preamble paragraph for informative discussion for the readers. It is important to compare and contrast your findings with those of others in the field. Take note of any similarities or differences, and provide plausible explanations for any discrepancies. Additionally, please review the last two paragraphs and provide clarification where needed. Begin by highlighting the strengths of this study, followed by any limitations that should be acknowledged.

7. Conclusions

Dear authors, please delete lines 349 to 354. Simply sate your major findings.

Decision: major revision

Thank you for your effort.
