# Supplementary material for: Screening of aspiration pneumonia using the modified Mallampati classification tool in older adults
Source: PLoS One. 2024 May 10;19(5):e0302384. doi: 10.1371/journal.pone.0302384 (PMC11086910; doi:10.1371/journal.pone.0302384)
Supplement: Attachment — Submitted filename: Dear Editorial Team.docx [file pone.0302384.s002.docx]

**Review of Manuscript**

- Title: Oropharyngeal morphology (modified Mallampati classification) could be a useful screening tool for the risk of pneumonia in the older adults
- Manuscript number PONE-D-23-40581
- Article type: Research Article

**Dear Editorial Team,**

- I sincerely appreciate you providing me the opportunity to review this significant scientific paper.
- I express my gratitude to the authors for their excellent work in examining the risk of pneumonia through non-invasive procedures, considering modifications in the morphology of the oropharynx and the position of the hyoid bone.

**General comments**

- The work could improve clinical practices and is crucial for the management of elderly patients.
- The writing is excellent, and the arrangement follows scientific guidelines.

**Specific comments**

1. Title: The title is informative and lucid.
2. Abstract: nicely put. However, the sampling strategy is left out. Add the sampling technique now.
3. Introduction: The writing is well done
4. Methods

- According to the authors, all research-related surveys were completed between May 16, 2021, and June 15, 2022. What makes the addition of one month (May 16, 2022 to June 15, 2022) noteworthy? The study took place between May 16, 2021 and May 15, 2022, if it was to last a full year.
- The study included 191 participants in total. What technique was used for sampling? The information was gathered at eleven hospitals. What percentage of participants was each hospital expected to contribute? Or was it just that any number of recruited subjects was employed in the research?
- The study included people who were 65 years of age or older as participants. Why should you use people who are 65 years of age or older? Why not sixty or fifty? or somebody else?
- Addition of dependent and independent variables was anticipated by the authors.
- Addition of operational definitions is necessary.

5. Results

- Well written

6. Discussion

- The findings of the study are well discussed with other studies.
