# Supplementary material for: Screening of aspiration pneumonia using the modified Mallampati classification tool in older adults
Source: PLoS One. 2024 May 10;19(5):e0302384. doi: 10.1371/journal.pone.0302384 (PMC11086910; doi:10.1371/journal.pone.0302384)
Supplement: Attachment — Submitted filename: Responce to Reviwers.docx [file pone.0302384.s003.docx]

Manuscript number **PONE-D-23-40581**

“**Screening of Aspiration Pneumonia using the Modified Mallampati Classification Tool in Older Adults**”

Thank you for reviewing our manuscript, which has now been revised in accordance with the editors’ and reviewers’ comments. Please find below our point-by-point response to the comments.

**1. Responses to the additional editor comments**

General basic Questions:

Q1.

**What specific criteria were used in the modified Mallampati classification to assess oropharyngeal morphology?**

A1.

Thank you for the question. We have added an explanation in the Materials and Methods section (Figure 1) (page 7; lines 110–113).

Q2.

**How was dysphagia assessed using the repetitive saliva swallowing test (RSST)? Can you provide more details about the RSST?**

A2.

Thank you for your suggestion. We have added an explanation in the Materials and Methods (page 6; lines 93–98).

Q3.

**Were there any specific inclusion or exclusion criteria for the recruitment of the 191 older participants?**

A3.

Thank you for your comment. We have added the recruitment criteria for the study participants in the Materials and Methods (page 5; lines 82–88).

Q4.

**What statistical methods were employed to analyze the data and determine the correlation between the modified Mallampati classification and aspiration pneumonia?**

A4.

Thank you for your comment. As shown in Tables 4 and 5, we used the Mann-Whitney U-test for the univariate analysis and logistic regression analysis for the multivariate analyses to investigate the correlation between the modified Mallampati classification and signs of aspiration.

Q5.

**Were there any limitations or potential confounding factors in the study design that could have influenced the results?**

A5.

Thank you for your comment. We have modified the limitations in the Discussion accordingly (page 27; lines 374–382).

Section specific questions:

Q6.

**Abstract: Could you provide more information about the specific methods used to assess oropharyngeal morphology and hyoid bone position? Additionally, please clarify the criteria used to define dysphagia and aspiration pneumonia.**

A6.

Thank you for the suggestion. We have added relevant explanations in the Abstract (page 2; lines 22–30).

Q7.

**Results: The correlation between the modified Mallampati classification and a medical history of aspiration pneumonia is mentioned. However, it would be helpful to include the strength of this correlation (e.g., correlation coefficient or p-value) to better understand the relationship.**

A7.

Thank you for your valuable comment. We have added the strength of the correlation in the Results (page 20; lines 280–281).

Q8.

**Results: The lack of significant association between the Mallampati classification and the repetitive saliva swallowing test (RSST) or history of choking/gag reflex is intriguing. Are there any potential explanations for this finding? If so, please discuss them.**

A8.

As mentioned in the Discussion (page 25-26; lines 337–360), choking or cough reflex from food and drinking is caused by swallowing problems. In addition, RSST may be mostly influenced by the muscular condition of the swallowing movements. Therefore, we hypothesized that the Mallampati classification is not associated with the dynamic state conditions of the patients but rather with their static state.

Q9.

**Discussion: It would be valuable to provide a more detailed discussion on the clinical implications of using the modified Mallampati classification as a screening tool for aspiration pneumonia. How might this classification be integrated into routine clinical practice? Are there any limitations or potential challenges in implementing this screening method?**

A9.

Thank you for your valuable suggestion. We have modified the last paragraph of the Discussion to further explain the integration of the Mallampati classification into routine practice (page 27; lines 383–386).

Q10.

**Funding Disclosure: The manuscript states that the authors received no specific funding for this work. However, it would be helpful to clarify whether there were any sources of funding that indirectly supported the study or the authors' involvement.**

A10.

Thank you for your query. There were no direct or indirect funding sources that supported this work.

Q11.

**Competing Interests: The authors have declared no competing interests. It would be beneficial to include a statement explaining that none of the authors have any financial or non-financial relationships that could be perceived as potentially influencing the interpretation of the results or biasing the study.**

A11.

None of the authors have any financial or non-financial relationships that can potentially influence the interpretation of the results or introduce bias in the study.

Q12.

**Ethics Statement: The manuscript mentions that the research was approved by the Ethics Committee. Please provide more details regarding the specific ethics approval, such as the name of the committee, the approval number, and any relevant ethical considerations that were addressed.**

A12.

Thank you for your comment. We have added the name of the committee, approval number, and all relevant ethical considerations in the last paragraph of the Materials and Methods (page 9-10; lines 154–160).

Q13.

**General: Please consider providing additional information regarding the demographic characteristics of the study participants, such as age range, gender distribution, and any relevant comorbidities. This information would help readers better understand the generalizability of the study findings.**

A13.

Thank you for your insightful comment. We have included details regarding the demographic characteristics of the study participants in Table 1.

Q14.

**Language and Clarity: Some sections of the manuscript could be further clarified to improve readability and understanding. I recommend a thorough proofreading to ensure accurate grammar, sentence structure, and scientific terminology throughout the manuscript.**

A14.

Thank you for your comment. We have had our manuscript re-checked by an English language proofreader.

Q15.

**Figures and Tables: Are there any figures or tables included in the manuscript to visually support the results or enhance the understanding of the study? If not, I suggest considering the inclusion of relevant visual aids to complement the text.**

A15.

Thank you for your suggestion. We have added Figure 4 as a visual aid to enhance the readers’ understanding of our study.

Q16.

**Please address these questions and comments in your revised manuscript.**

A16.

Thank you for your comments regarding our study. We have implemented all the requested changes in the revised manuscript.

**Editor Comments:**
1.

**The abstract provides a clear overview of the study objectives and methods.**

A.

Thank you for your valuable comment.

2.

**The results indicating a significant correlation between the modified Mallampati classification and a medical history of aspiration pneumonia are interesting. However, it would be helpful to discuss the clinical implications of this finding in more detail.**

A.

Thank you for your comment. We have discussed the clinical implications of this finding in more detail in the Discussion.

3.

**It would be beneficial to include a discussion on the potential applications of the modified Mallampati classification as a screening tool for dysphagia and aspiration pneumonia in clinical practice.**

A.

Thank you for your comment. We have added the potential applications of the modified Mallampati classification as a screening tool for dysphagia and aspiration pneumonia in clinical practice in the Discussion (page 27; lines 383–386).

4.

**Providing additional information on the demographic characteristics of the study participants, such as age distribution and gender ratio, would enhance the understanding of the study population.**

A.

Thank you for your comment. We have included details regarding the demographic characteristics of the study participants in Table 1.

5.

**Consider discussing the limitations of using self-reported history of choking or coughing reflex as a measure of aspiration signs, as this may introduce recall bias.**

A.

Thank you for your suggestion. We have explained the limitation of this study in terms of the possibility of recall bias in the self-reported information (page 27; lines 378–379).

**Responses to reviewer comments**

Reviewer 1

Q1.

**Title; The title should be like “Screening of Aspiration Pneumonia using the Modified Mallampati Classification Tool in Older Adults”.**

A1

Thank you for your helpful suggestion. We have changed the title to “**Screening of Aspiration Pneumonia using the Modified Mallampati Classification Tool in Older Adults**” accordingly.

Q2

**The introduction part of the abstract should be rewrited. It is so long. Make it short and concise. From lines 19 to 21 is not clear: “We hypothesized that changes in the oropharyngeal morphology and hyoid bone position may lead to the evaluation of swallowing function, which in turn determines the risk of aspiration pneumonia”. Please rewrite it as ‘Changes in the oropharyngeal morphology and hyoid bone position may lead to the risk of aspiration pneumonia’.Lines 30-32: “The lower the classification (small size of the base of the tongue relative to the oropharyngeal cavity), the higher was the incidence of aspiration pneumonia.” This statement is not clear. Please rephrase or delete it.**

A2

Thank you for your feedback. We have modified the abstract for better flow and readability as per your advice.

Q3.

Introduction

**Dear authors, in the introduction section, lines 38 to 43 have no references. This is not acceptable in a scientific work. Please add citations in each statement unless interlinked. Similarly lines 47 to 63 have no references. Dear authors, you only cite 2 references in the whole section of the introduction. Please take more time, you have to cite every piece of evidence that you wrote. Please rephrase each paragraph. It needs English language revision. For instance, lines 45 to 46: “Thus, there is an urgent need to manage aspiration pneumonia in an aging society.” Should be moved to the last paragraph of the ‘introduction’ section.**

A3.

Thank you for your suggestions. We have rephrased the specified sentences in the Introduction and added the corresponding references.

Q4.

Methods

**Dear authors, please add study area/setting, study period and sample size calculation sub-headings. Please move ethical declaration statements with sub heading “ethical approval” (Lines 71 to 76) to the end of ‘methods’ section or at the end of the manuscript before the ‘references’ section. Please separately state your inclusion and exclusion criteria. I think you need to add “operational definition” subheading. For example: menton, laryngeal prominence and validity and reliability of your measurements. Please consult native speaker of English language for grammar and punctuation errors.**

A4.

Thank you for your comments. We have added subheadings for the study area/ sampling and ethical approval subsections. Additionally, we have rephrased the inclusion and exclusion criteria. Further, we have added an explanation for each measurement to make the operational definition easier to understand.

Q5.

**Results**

**Dear authors, no need of this statement “The characteristics of the participants are presented in Table 1. ”Instead please cite table 1 in parenthesis at the end of this paragraph. Lines 140 to 141 should be rewrite as ‘The median age of participants (men: 92, women: 99) was 79.3 years with a range of 65 to 98 years’.Dear authors, please rewrite lines 142 to 144 as ‘About 152 (79.6%) participants had been evaluated for saliva swallowing tests more two times.’ Please continue like this …..No need of writing lower frequencies or percentages in the table. Focus on pertinent findings.**

**Dear authors please delete lines 247 to 248 and cite table 5 in parentheses at the end of this paragraph.**

A5.

Thank you for your insightful comments. We have modified the specified sentences as proposed.

Q6.

**Discussions**

**Dear authors, please delete “We believe that this is the first study to examine the relationship between the Mallampati classification and the risk of aspiration pneumonia”.No need of this statement rather if possible it is better to add a preamble paragraph for informative discussion for the readers.It is important to compare and contrast your findings with those of others in the field. Take note of any similarities or differences, and provide plausible explanations for any discrepancies. Additionally, please review the last two paragraphs and provide clarification where needed. Begin by highlighting the strengths of this study, followed by any limitations that should be acknowledged.**

A6.

Thank you for your valuable comments. We have modified the Discussion as proposed.

Q7.

**Conclusions**

**Dear authors, please delete lines 349 to 354. Simply sate your major findings.**

A7.

Thank you for your suggestion. We have modified the sentences accordingly.

**Reviewer 2**

Q1.

**Title: The title is informative and lucid.**

A1.

Thank you for your comment.

Q2.

**Abstract: nicely put. However, the sampling strategy is left out. Add the sampling technique now.**

A2

Thank you for your suggestion. We have added the sampling method.

Q3.

**Introduction: The writing is well done.**

A3.

Thank you for your comment.

Q4

**All research-related surveys were completed between May 16, 2021, and June 15, 2022. What makes the addition of one month (May 16, 2022 to June 15, 2022) noteworthy? The study took place between May 16, 2021 and May 15, 2022, if it was to last a full year.**

A4

Thank you for providing these insights. We apologize for any confusion. Sampling was conducted over a period of 13 months in the multicenter survey.

Q5

**The study included 191 participants in total. What technique was used for sampling? The information was gathered at eleven hospitals. What percentage of participants was each hospital expected to contribute? Or was it just that any number of recruited subjects was employed in the research?**

A5

Thank you for the query. For this survey, the examiners in each hospital randomly recruited patients from potential individuals who met the inclusion criteria as mentioned in the Materials and Methods using a simple random sampling method. Regarding the number of patients, there was not an expected percentage of participants from each hospital.

Q6.

**The study included people who were 65 years of age or older as participants. Why should you use people who are 65 years of age or older? Why not sixty or fifty? or somebody else?**

A6.

Thank you for your comment. In this study, we aimed to investigate a screening tool for identifying the risk of pneumonia especially in older adults. Additionally, in Japan, 65 years of age or older is widely defined as older adults in the social welfare systems.

Q7.

**Addition of dependent and independent variables was anticipated by the authors.**

A7.

Thank you for your suggestion. We have added the dependent and independent variables.

Q8.

**Addition of operational definitions is necessary.**

A8.

Thank you for your comment. We have added the operational definitions as suggested (page 6,7 ; lines 93–98, 110-113).

Q9.

**Results
Well written.**

A9.

Thank you for your comment.

Q10.

**Discussion**

**The findings of the study are well discussed with other studies.**

A10.

Thank you for your comment.
